# A Study of Laser Micromachining of PM Processed Ti Compact for Dental Implants Applications

**DOI:** 10.3390/ma12142246

**Published:** 2019-07-12

**Authors:** Peter Šugár, Jaroslav Kováčik, Jana Šugárová, Barbora Ludrovcová

**Affiliations:** 1 Faculty of Materials Science and Technology, Slovak University of Technology, J. Bottu 25, 917 24 Trnava, Slovakia; 2 Slovak Academy of Sciences, Institute of Materials and Machine Mechanics, Dúbravská cesta 9, 845 13 Bratislava, Slovakia

**Keywords:** laser, machining, titanium, powder metallurgy, implant, surface morphology

## Abstract

The paper deals with the experimental study of laser beam micromachining of the powder metallurgy processed Ti compacts applying the industrial grade fibre nanosecond laser operating at the wavelength of 1064 nm. The influence of the laser energy density on the surface roughness, surface morphology and surface elements composition was investigated and evaluated by means of surface roughness measurement, scanning electron microscopy (SEM), energy dispersive X-Ray spectroscopy (EDS) and X-ray diffraction (XRD) analysis. The different laser treatment parameters resulted in the surfaces of very different characteristics of the newly developed biocompatible material prepared by advanced low temperature technology of hydride dehydride (HDH) titanium powder compactation. The results indicate that the laser pulse energy has remarkable effects on the machined surface characteristics which are discussed from the point of view of application in dental implantology.

## 1. Introduction

Titanium and titanium-based alloys find a wide-range engineering applications in many industrial areas owing to their excellent mechanical and chemical characteristics, high strength to weight ratio, good corrosion resistance, outstanding biocompatibility, relatively low density (4.5 g·cm^−3^) and low Young’s modulus [1,2,3]. As biomedical implants, they are used in dental and orthopaedic fields, as bone plating, screws and hard tissue replacements [4,5,6,7]. Some surface micro-morphology modification technologies are used to improve biocompatibility and enhance osseointegration of the titanium implants. Osseointegration—the structural linkage made at the contact point where the human bone and the surface of an implant meet—is supported by the implant surface porosity, surface roughness or by regular surface patterns (texture) with texture elements smaller than 100 μm [8,9]. These structures may be fabricated by different technologies, such as sintering, sandblasting [10], chemical etching [11], plasma spraying [12], electrical discharge machining [13], electron beam texturing etc., including laser beam micro-machining [14,15,16,17].

By the laser micro-machining, a material is ablated from surface by a pulsed laser beam of high energy density. Material evaporation from the surface forms cavities in the irradiated area, while increasing roughness of the machined surface. The interaction between the material and the laser beam is influenced by the properties of the machined material and the input process parameters. Complex surface structures can be formed by optimizing theses parameters. [18,19,20,21,22,23,24,25,26,27,28,29,30].

Erdogan et al. [27] showed that high-precision and repeatability was achieved by the femtosecond and picosecond fibre lasers texturing of the titanium implant surfaces at 1 MHz and 43 MHz repetition rate. Celen et al. [28] created 3D patterns on cp Ti, using the ytterbium fibre laser (20 W, 1064 nm, 125 kHz, 200–250 ns pulse durations) and using laser pulse intensities of approximately 13.92 × 10^8^ W·cm^−2^. Shinonaga et al. [29] investigated the influence of the pulse widths (from 200 fs to 800 fs) on the nanostructures formation on a Ti plate using femtosecond laser (790 nm) at the laser intensity of 2.1 × 10^12^ W·cm^−2^ (laser fluence 0.35 J·cm^−2^, track displacement 55 µm). Wang et al. [30] created micro-patterns with deposited calcium/phosphorus (Ca/P) elements on a pure titanium surface by a femtosecond laser (800 nm, 1 kHz). The gradual increase of laser fluence to 3.3 J·cm^−2^, 6.6 J·cm^−2^ and 12.5 J·cm^−2^ results in the larger holes and islands sizes of 0.5 μm and 2 μm to 5 μm and 6 μm. Oliveira et al. [31] also used a femtosecond laser (pulse duration 500 fs, wavelength 1030 nm) for cp Ti grade 2 machining. The ripples were observed on the periphery of the irradiated area, whenever the radiation fluence was lower than the threshold. Serkov et al. [32] ablated a bulk Ti target by 10 ps laser pulses in water. The titanium oxide nanoparticles observed on the machined surface improved its corrosion resistance. Pfleging et al. [33] achieved periodic lines and dimple patterns without defects on Ti–6Al–4V by using an ArF excimer laser (wavelength 193 nm, pulse duration 5 ns). Many authors examined the surface roughness after laser texturing to improve mechanical properties of the bone-implant interface and stress distribution. Less strong bone responses were found by the smooth (Sa < 0.5 µm) and minimally rough (Sa 0.5–1 µm) surfaces of implants. Moderately rough (Sa > 1–2 µm) surfaces showed stronger bone responses than rough ones (Sa > 2 µm) [15]. Radmanesh et al. [34] increased surface roughness and oxidation by applying a nanosecond pulsed laser (frequency 25 kHz, power 7 W, scanning speed 100 µm·ms^−1^, 300 µm·ms^−1^, 500 µm·ms^−1^). Chikarakara et al. [35] investigated the effects of the high speed laser surface modification of Ti–6Al–4V. The measured average roughness of the grit blasted alloy was 0.56 ± 0.1 μm. The laser surface processing subsequently produced average roughness values between 1.39 μm and 2.73 μm. Worts et al. [36] used a femtosecond laser (200 W, wavelength 1040 nm) for machining the Ti–6Al–4V alloy powder particles. Final surface roughness value Ra of 0.8 μm was achieved by processing the material in a single pass in a raster pattern. Hsiao et al. [37] increased surface roughness of Ti–6Al–4V using a low energy pulsed ultraviolet (UV) laser (wavelength 355 nm, output power 14 W at 100 kHz). The UV laser texturing protocol resulted in a mean surface roughness Ra of 0.755 μm compared to a Ra of 0.556 μm for the machined surfaces. Thereafter, a subset of three HA-coated implants was treated by the UV laser resulting in a mean surface roughness of 3.3 μm. Lee et al. [38] reported the higher mean roughness values with the sand-blasting and acid-etching technique used to treat the surface of cp Ti (1.285 ± 0.025 μm). When adding microgrooves made by a laser, the investigators obtained a mean roughness of 22.35 ± 2.76 μm. 

Based on the studies, it is clear that the research on biocompatible materials and their processing with the goal of optimizing the topographical features of the functional surfaces of implants is still of outmost importance since many questions concerning the optimal surface morphology have not been answered yet. This fact, combined with the growing interest in the use of Ti powder metallurgy (PM) as a cost-effective way of direct production of complex parts made of Ti and its alloys [39], has led the authors to study the laser micro-machining of Ti samples, prepared by a pioneering low temperature powder metallurgy technique. The influence of the laser energy on the surface morphology and roughness was investigated and evaluated in the research described in this study, and discussed regarding its application in dental implantology. 

## 2. Experimental

### 2.1. Experimental Material

**Figure 1:**
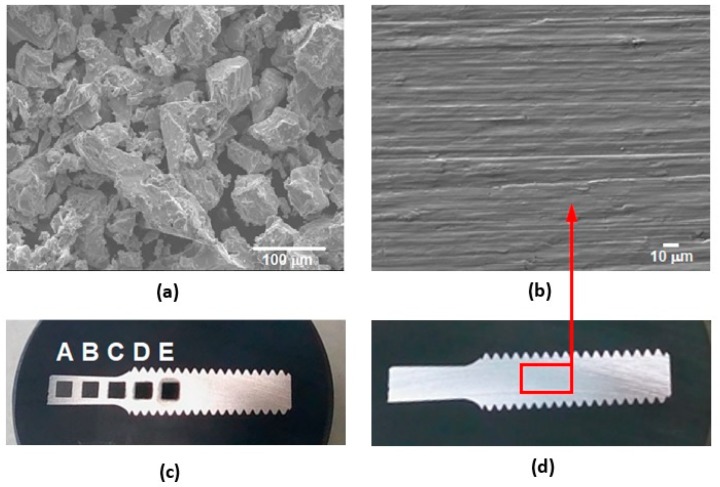
Experimental sample: (**a**) scanning electron microscopy (SEM) image showing a characteristic fragmented shape of the used HDH titanium powder, original magnification of 250×, (**b**) SEM image of the sample surface before machining, original magnification of 500×, (**c**) experimental sample after laser machining, (**d**) experimental sample before laser machining.

The material used in the experiment was a titanium powder compact made from the HDH (hydride-dehydride) titanium powder of the particle size below 150 μm and oxygen content 0.21 ± 0.01 wt.% (Kimet Special Metal Precision Casting Co., Ltd., Hengshui, China). The powder is of a typical fragmented shape, owing to the HDH preparation method (see Figure 1a). The powder size distribution was determined using Fritch Analysette 22 (FRITSCH GmBH – Milling and Sizing, Weimar, Germany) laboratory equipment and wet dispersion. The obtained results were d_50_ = 29 μm and d_90_ = 97 μm. The experimental material was made by cold isostatic pressing (CIP) at 200 MPa followed by preheating of the green compact of the porosity between 35–42% in a furnace in the air at 450 °C for 30 min and compacted by the direct extrusion (DE) into the 6 mm-diameter rods at the temperature of 500 °C. DE was conducted by using a 180° nozzle die with an area reduction ratio of 11:1 and a ram speed of 0.6 mm·s^−1^. The microstructure of low temperature PM processed Ti compacts consists of α titanium phase and residual porosity of theoretical density (THD) of 99.1% [40,41]. Finally, the Ti sample was prepared by wire electrical discharge machining (WEDM) followed by mechanical grinding by abrasion paper of fineness 1200, and ultrasonic cleaning. The final surface roughness Ra of 0.3 ± 0.03 μm was obtained.

**Figure 2:**
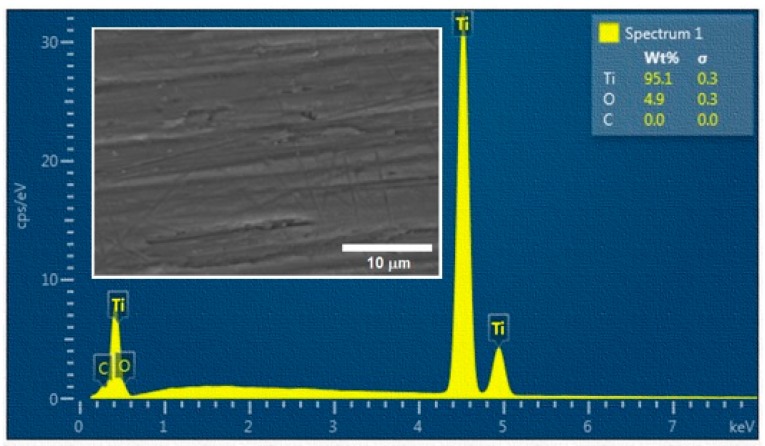
Result of energy dispersive X-Ray spectroscopy (EDS) analysis of a non-irradiated surface.

The scanning electron microscopy (SEM) image of the base material and the result of the energy dispersive X-Ray spectroscopy (EDS) analysis are shown in Figure 1b and Figure 2, respectively. Mechanical properties of the low temperature PM processed titanium compact and THD compared with the properties of CP Ti Grade 1 are depicted in Table 1. It shows the decrease in modulus of elasticity (E) and elongation to fracture (A_t_) accompanied by a significant increase of strength properties (yield stress R_p0.2_, ultimate tensile strength R_m_) of the prepared Ti samples.

### 2.2. Experimental Methods

**Figure 3:**
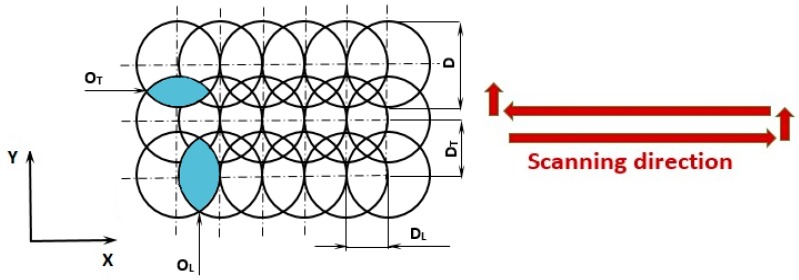
Schematic representation of the laser beam tracks. D—spot diameter, D_L_—lateral overlap distance, D_T_—transverse overlap distance, O_L_—lateral overlap, O_T_—transverse overlap.

In the research, the machining centre of Lasertec 80 Shape equipped with the nanosecond fibre laser system was employed for ablation of cavities of a square shape and a side 1.5 mm long. Through changing the laser pulse energy (E_p_) in the range of 0.2–1 mJ, with the increment of 0.2 mJ, five cavities labeled as A, B, C, D and E with different types of surfaces were obtained. The five replications of the experiment were used in this study. During the experiment, a constant pulse frequency of 20 kHz, scanning speed of 100 mm·s^−1^ and a laser spot diameter of 50 μm were set up. A uni-directional traces layout (hatching strategy) was performed with a lateral (O_L_) and transverse (O_T_) pulse overlap of 90% and 50%, respectively (Figure 3). The material was ablated in one layer under the ambient air conditions in order to generate the oxidation of the machined surface.

In order to assess the effects caused by laser ablation of the PM processed Ti samples, several procedures were developed. Roughness parameters were measured using a contact-gauge Zeiss Surfcom 5000 profilometer. The profile parameter Ra (arithmetical mean height), Rz (maximum height of profile), the Abbott-Firestone curve parameters Rpk (reduced peak height) and Rvk (reduced valley depth) were elaborated and reported. The roughness was evaluated on each machined surface with repetition 5 times. Further, surface morphologies were investigated using a scanning electron microscope (SEM) JEOL JSM 7600F (JEOL Ltd., Tokyo, Japan) with resolution of 1.5 nm (1 kV) in a gentle beam mode and 1.0 nm in 15 kV; magnification from 25 to 1 000 000 times. The energy dispersive X-ray spectrometry (EDS) was conducted to evaluate chemical composition of the machined surfaces by an EDS analyser integrated in the SEM. Finally, the indications of oxides on the substrate material by the X-ray diffraction (XRD) measurement using a Brucker D8 difractometer and a Brucker D8 difractometer with rotating anode (Brucker, Billerica, MA, USA) were performed.

For statistical evaluation of the surface roughness parameters of each machined surface, the one-way analysis of variance (ANOVA) and Tukey post-hoc test for multiple comparisons between the groups were performed. The tests were carried out at the significance level of 0.05, applying Minitab version 17 Software (Minitab, LLC, State College, PA, USA).

## 3. Results

**Figure 4:**
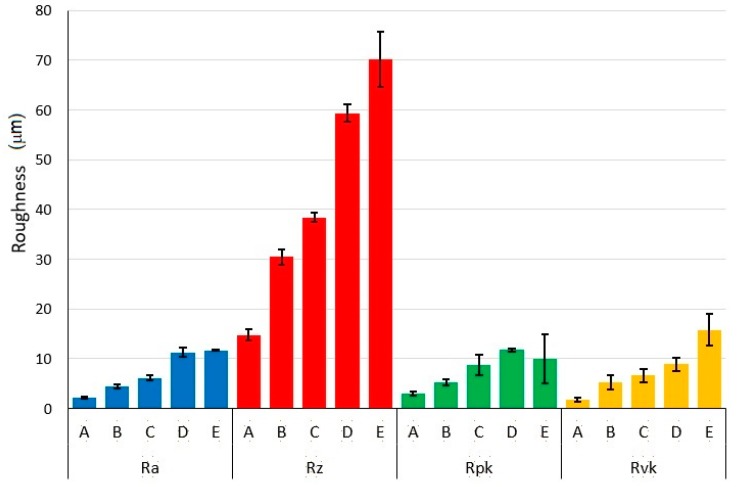
Roughness development depending on applied pulse energies.

The results of the amplitude surface roughness parameters of Ra, Rz and the bearing curve parameters of Rpk and Rvk of non-irradiated surface and surfaces ablated under different laser beam energy conditions are summarized in the Table 2 and Figure 4. They bring the means and standard deviations (SD) of the roughness parameters evaluated in Y direction, perpendicular to the direction of the beam motion. As a general trend, the surface roughness increased with higher pulse energy.

**Figure 5:**
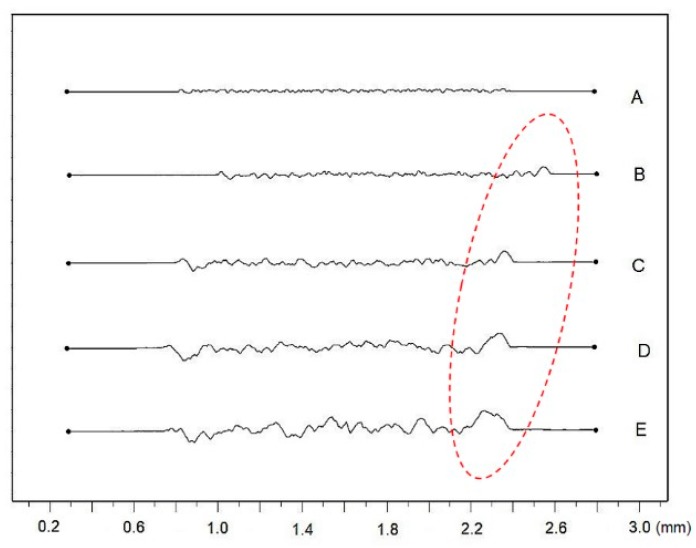
Comparisons of the machined surface profiles with indicated moved molten material.

**Figure 6:**
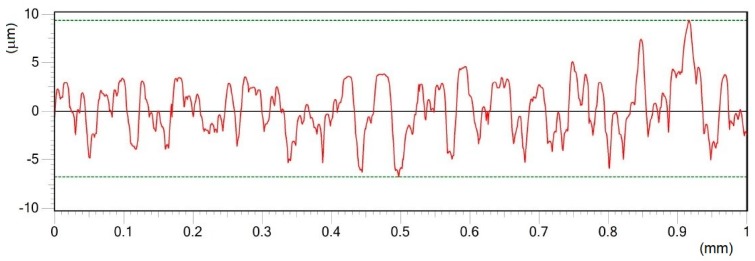
Roughness profile of surface A in Y direction treated by pulse energy of 0.2 mJ; Ra 2.18 μm.

**Figure 7:**
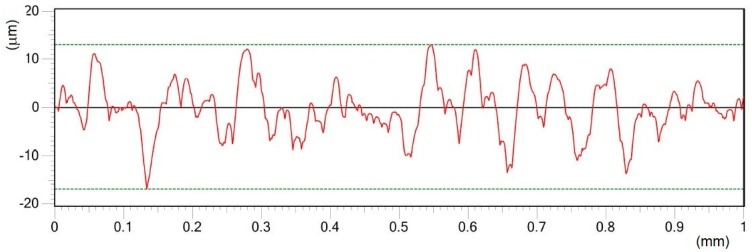
Roughness profile of surface B in Y direction treated by pulse energy of 0.4 mJ; Ra 4.37 μm.

**Figure 8:**
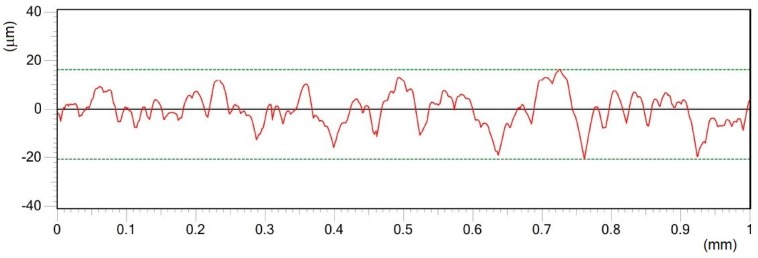
Roughness profile of surface C in Y direction treated by pulse energy of 0.6 mJ; Ra 6.15 μm.

**Figure 9:**
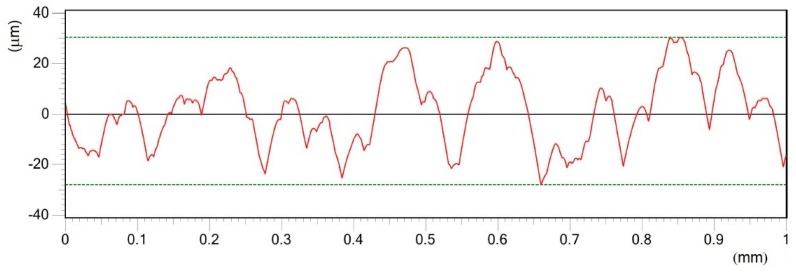
Roughness profile of surface D in Y direction treated by pulse energy of 0.8 mJ; Ra 11.43 μm.

**Figure 10:**
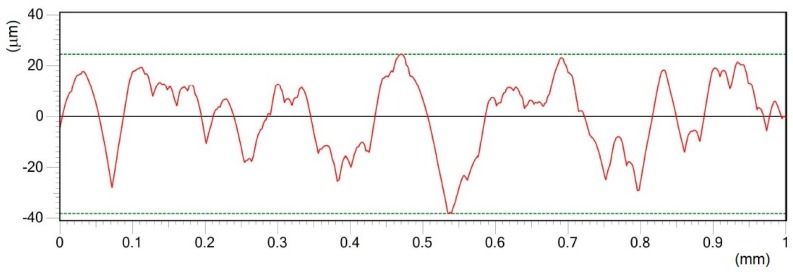
Roughness profile of surface E in Y direction, treated by pulse energy of 1 mJ; Ra 11.73 μm.

Roughness profiles of the machined surfaces are documented in Figure 5, Figure 6, Figure 7, Figure 8, Figure 9 and Figure 10. From an examination of the surface morphology, it is possible to note the loss of uniformity of the surface profile when the higher pulse energies are applied. It is the result of the massive melting phenomena involving all the zones exposed to the laser. The established random solidification front leads to the displacement of a considerable amount of molten material (see right side of full profiles in Figure 5) over the laser treated substrate, and the establishment of a completely altered surface morphology.

**Figure 11:**
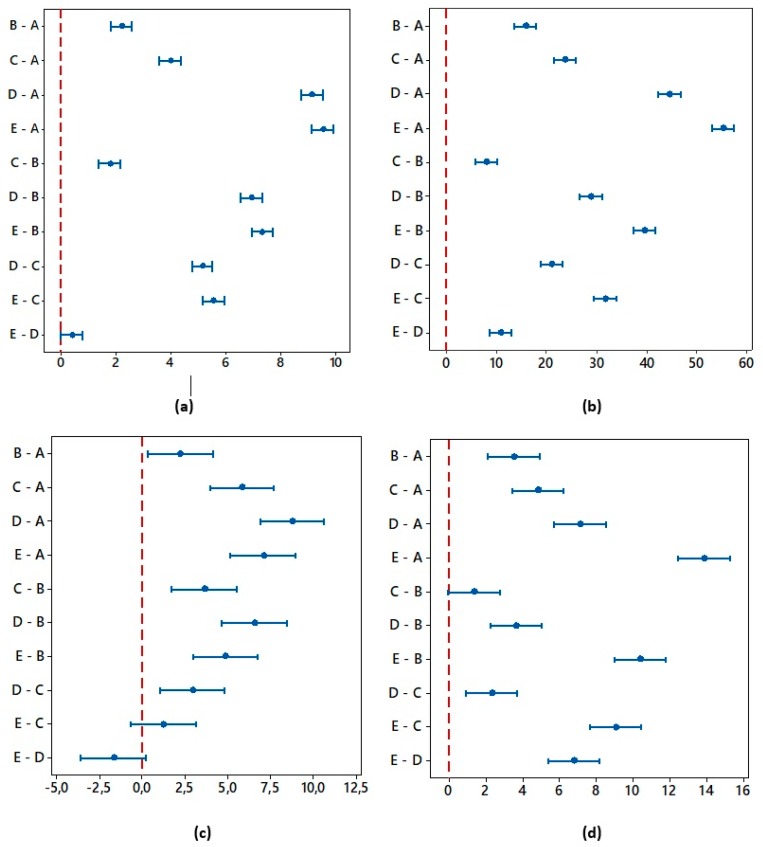
Tukey post-hoc test for (**a**) Ra, (**b**) Rz, (**c**) Rpk, (**d**) Rvk.

The one-way ANOVA (Fisher’s test) results are reported in the Table 3. For all roughness parameters the alternative hypothesis was confirmed, i. e., at least one mean value of Ra, Rz, Rpk and Rvk exhibits a difference on the significance level of α = 0.05 and α = 0.01. The results of Tukey post-hoc test, identifying statistically significant differences between specific groups are documented in Figure 11. Basically, the tests for Ra and Rpk indicate that similar roughness results were observed for the pairs of surfaces D and E with high laser pulse energies, which is also confirmed by microstructure of the surfaces.

**Figure 12:**
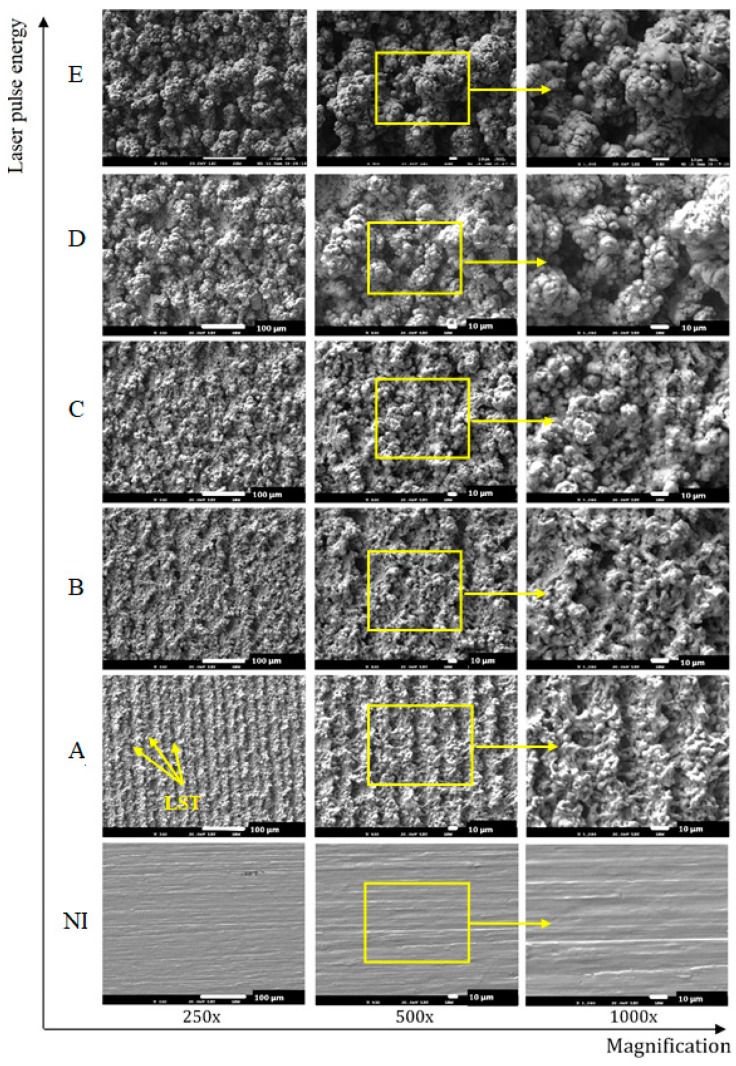
SEM images of the machined surfaces, showing the influence of the fluence on the surface morphology: NI—non-irradiated surface (Ra 0.25 μm); Surface A (E_p_ = 0.2 mJ, Ra 2.95 μm); Surface B (E_p_ = 0.4 mJ, Ra 4.71 μm); Surface C (E_p_ = 0.6 mJ, Ra 6.38 μm), Surface D (E_p_ = 0.8 mJ, Ra 9.56 μm), Surface E (E_p_ = 1 mJ, Ra 11.67 μm); LST - Laser Scanning Tracks.

The results of SEM observations of the machined surfaces acquired at three different levels of magnification (250×, 500× and 1000×) are shown in Figure 12. The morphologies resulting from the fast melting and solidification process were identified on all sample surfaces. The morphology significantly varies depending on the increased laser pulse energy. The higher values of energies correspond to a rougher machined surface. While at low laser pulse energy, the laser scanning tracks separated by 25 microns were clearly visible, at higher laser pulse energies, they disappeared and no surface texture was observed.

**Figure 13:**
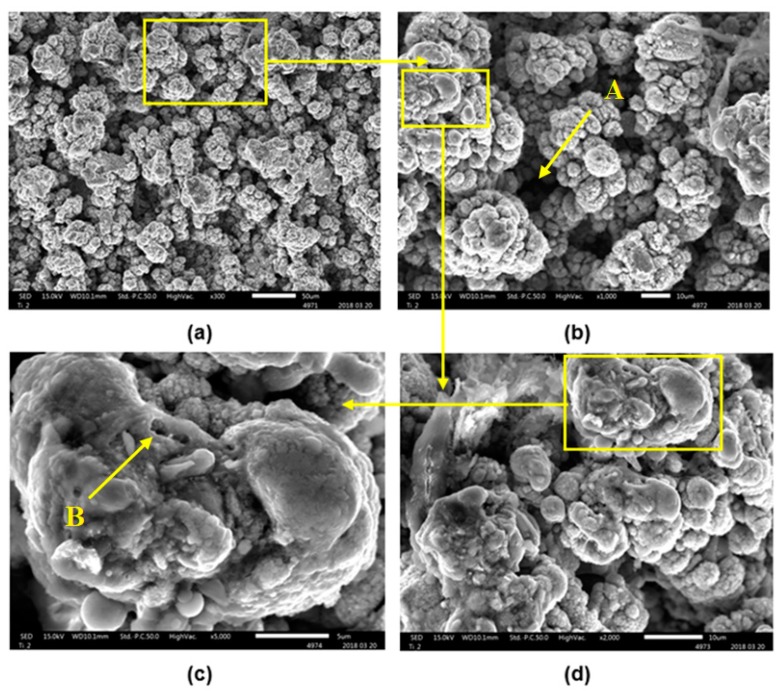
Multiple SEM micrographs of the machined surface B: (**a**) Original magnification of 300×, (**b**) original magnification of 1000×, (**c**) original magnification of 5000×, (**d**) original magnification of 2000×; A—macro-sized pores, B—micro-sized pores.

A more detailed view of the machined surface B, using magnifications 2000× and 5000×, are seen in Figure 13. The sample exhibits a rough surface consisting mainly of the spheroidized and partially melted particles, as well as of some melted and flat areas. Ti particles are found to be partially melted on the surface. These surface melted particles join together owing to the presence of liquid metal at the particle interfaces and bond well with the previous layers.

During laser machining, not only significant changes of surface morphology were observed, but an important change in the surface chemistry was induced as well. The increase of the pulse energy was accompanied by the increase of the oxygen content in the machined surface, as the experiments were performed under ambient air conditions. The results of EDS area analysis obtained from evaluation of three surfaces randomly selected from the set of five replications of the experiment are depicted in Table 4.

**Figure 14:**
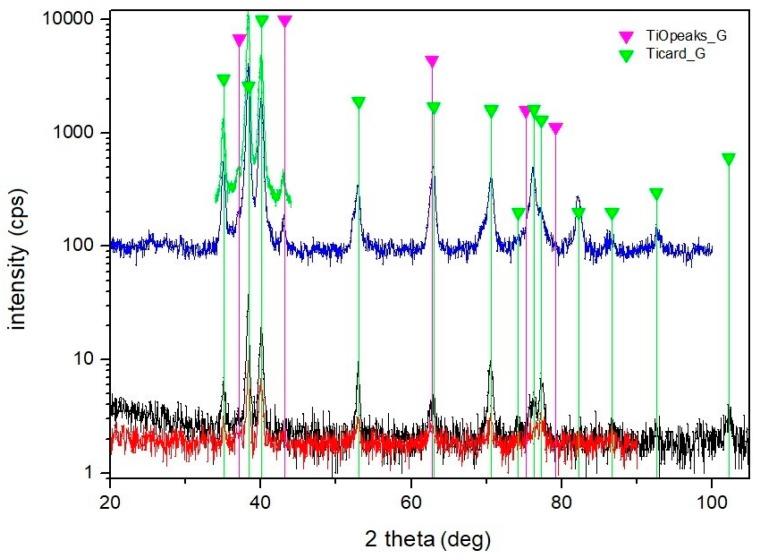
X-ray diffraction patterns of machined surface E (Brucker D8, incidence angle 10 deg {black colour) and incidence angle 15 deg (red colour); Brucker D8 with rotational anode, incidence angle 6 deg (blue colour) and 12 deg (green colour).

The result of XRD analysis of machined surface E is depicted in Figure 14. It is evident that the Ti and TiO peaks are visible in the difractograms. All the diffraction peaks are well matched with the International Centre for Diffraction Data (ICDD) reference cards n° 00-044-1294 (titanium) and n°. 01-089-5010 (TiO).

## 4. Discussion

The success of clinical application of titanium implants strongly depends on several factors, including the implant surface properties, such as surface macro-, micro-, and nano-topography, chemical composition, wettability, hydrophilicity and hydrophobicity which all influence the reaction of the host tissue, lengthen the implant’s life, increase its performance and improve the attachment of the implant with the biological tissue and bone. The implant surface morphology plays a very important role in the bone healing process. Further, it was confirmed that moderately rough surfaces, compared with the smoother or rough surfaces, have the best effect on the osteoblast differentiation and migration [43,44].

The bone in-growing is effectively influenced when the implant of porous surface is used, since the pores and porous structures improve biocompatibility by attaching cells to the porous structure of the implant [16]. The surfaces of the topographies rough at a micrometer scale, periodic surface textures and surfaces with grooves, micro pits, scratch marks and pores of different sizes are being intensively studied and tested. However, at present, the optimum topography and surface roughness for dental implants are still being researched and investigated. Studies have shown the positive impact of the surfaces, containing pits, grooves and protrusions, on the positive biological responses at the bone-to-implant interface. It is due to the increased surface area at the bone-to-implant interface. [45].

Shah et al. [46] observed osteocytes aligned adjacent to the machined implant surface, but the no osteocyte canaliculi (Ot.Ca) directly attached to the machined implant surface. On the other hand, canaliculi were found in high numbers adjacent to the laser-modified implant surface. These canaliculi appeared to branch, thus forming an extensive intercommunicating network closely adhering to the complex microtopography of the laser-ablated areas. Walker et al. [47] observed in the case of the Ti-6Al-4V samples manufactured by selective laser melting, that the fatigue life was controlled by a combination of the initiating defects (typically lack of fusion defects and/or porosity) and inherent variability in the fatigue crack growth rate characteristics of the material produced by selective laser melting. Moreover, they further observed that the fatigue performance for samples manufactured by selective laser melting without post-processing was significantly inferior to conventionally manufactured material. A similar situation concerning osteogenesis behavior and fatigue life can be probably observed for Ti implants prepared by powder metallurgy (small residual porosity and crack growth from the laser treated surface).

The surface topography and surface chemistry of the low temperature PM processed titanium compact after nanosecond laser treatment, evaluated in this study, showed that the thermal energy accumulated on the surface has a significant impact on the machined surface topography, roughness and surface layers composition. The pulse energies in the range of 0.2–1 mJ employed in this investigation revealed the surfaces roughness Ra in the range of 2.18–11.7 μm. The SEM analysis of the examined surfaces indicated that increased laser pulse energy was associated with an increase of the ablation depth (Rz up to 70.16 μm). The surface topography was formed of the ridges of the molten redeposited and solidified titanium globules, craters and voids. It contained agglomerated particles of solidified titanium (Figure 13) combined with the irregularly shaped macro and micro pores of the sizes between 10–25 μm and 1–3 μm, respectively. The typical microscale irregularities, having resulted from the melting and partial melting of the machined material, are visible. Being responsible for the cellular bioactivity and improves the implantation performance, these phenomena are desirable owing to the improvement of the adhesion between the bone tissue and the implant [48,49]. The lower amount of energy used in the laser surface treatment resulted in a typical surface topography with visible laser beam traces which did not occur when higher laser pulse energies were used.

The EDS and XRD analyses of the non-irradiated and laser treated surfaces in ambient air confirmed the existence of oxidation of titanium, which was the result of the instantaneous energy effect, and the content of O which came from the surrounding environment. The content of O increased significantly with the increasing laser beam pulse energy. On the other hand, it can be stated that only TiO was detected by the X-ray diffraction. The thickness of the oxide layer was very thin and it was hardly detected. Surprisingly, no TiO_2_ was observed on the laser treated surface, even for sample E with highest input of laser energy. TiO_2_, as the most thermodynamically stable form of the oxide, was probably contained in an amorphous structure which was formed due to the very rapid cooling of liquid metal. The analysed surfaces did not show any other contamination.

It is evident from the conducted experiments that the techniques of powder metallurgy combined with the laser surface micromachining provide a cost-effective alternative applicable in dental implantology. This is mainly owing to their ability to generate surfaces with specific porosity which improves the anchoring between the implant and bone, and stimulates a better functional and structural bond. If taking into account the fact that surface roughness Ra of dental implants is in the range of 1–10 μm, and roughness of the majority of the up-to-date dental implants is Ra of 1–2 μm [45], the lower level of pulse energy is recommended for the optimal surface treatment of the studied PM titanium compact.

## 5. Conclusion

In this research, the low-temperature powder metallurgy processed Ti was treated by applying different energies of the laser beam. It resulted in different quality of surface finishes, which is discussed in relation to early osseointegration of dental implants. Based on the results of the experimental investigation and statistical analysis, the following conclusions might be drawn:

(1) Different processing laser pulse energies were confirmed to have a great effect on the qualities of machined surfaces. It was observed that higher laser fluences lead to a rougher surface finish. The surfaces of porous-like appearances were revealed after laser treatment for every used pulse energy level.
(2) The treated surface is formed of ridges of the molten and solidified titanium globules, craters and voids. It contains agglomerated particles with the irregular macro- and micro- pores of sizes of 10–25 μm and 1–3 μm, respectively.
(3) The results of the one-way ANOVA analysis brought an overall statistically significant difference in the group means for all roughness evaluated parameters. The main differences between the surfaces A and E were confirmed by the Tukey post-hoc test.
(4) Owing to the treatment in ambient air, the oxidation of titanium took place. With the increase of the laser beam pulse energy, the content of O increased significantly.
(5) This study helps to identify the laser beam energy parameters for achieving a pre-defined surface geometry. The lower level of pulse energy is recommended for the optimal surface treatment of the studied PM titanium compact, when the large lateral pulse overlap is applied.
(6) Recorded surface roughness parameters of laser treated Ti powder compact produced at low temperatures provide good conditions for applications in the field of dental surgery.
(7) However, the contribution brings only partial insights into an otherwise wide problem, so for a wider application of the studied material, it is necessary to carry out a further series of experiments, especially focused on bio-testing.

## Figures and Tables

**Table 1:** Mechanical properties of the low temperature PM processed titanium compacts compared with the properties of the CP Ti Grade 1 [42].

Material	THD (%)	E (GPa)	R_p0.2_ (MPa)	R_m_ (MPa)	A_t_ (%)
**Ti compact**	99.13	94.5	541	686.7	4.08
**CP Ti Grade 1**	100	105	170-310	240	24

**Table 2:** The mean and standard deviations of the amplitude roughness parameters.

Surface	N	Ra (μm)		Rz (μm)		Rpk (μm)		Rvk (μm)	
		Mean	SD	Mean	SD	Mean	SD	Mean	SD
A	25	2.18	0.20	14.80	1.15	2.97	0.37	1.78	0.35
B	25	4.38	0.38	30.48	1.51	5.19	0.58	5.31	1.45
C	25	6.16	0.50	38.39	0.97	8.80	2.06	6.62	1.36
D	25	11.31	0.86	59.35	1.73	11.73	0.34	8.92	1.35
E	25	11.70	0.18	70.16	5.61	10.05	4.92	15.68	3.21

**Table 3:** One-Way ANOVA.

Roughness Parameter	DF1	DF2	F-value	*p*-value	R^2^
Ra	4	120	1865.18	0.000 *	98.42
Rz	4	120	1582.94	0.000 *	98.14
Rpk	4	120	55.5	0.000 *	64.19
Rvk	4	120	206.65	0.000 *	87.32

* At least one mean is different for significance levels of α = 0.05 and α = 0.01.

**Table 4:** The result of the EDS area analysis.

Surface	N	Pulse Energy (mJ)	wt. % of Elements	
			Ti	O
NI	3	–	93.9 ± 1.1	5.5 ± 0.7
A	3	0.2	80.4 ± 0.9	21.5 ± 0.9
B	3	0.4	72.6 ± 1.1	28.5 ± 1.2
C	3	0.6	67.9 ± 0.6	31.7 ± 0.9
D	3	0.8	61.8 ± 1.3	34.6 ± 2.8
E	3	1	58.8 ± 1.6	37,9 ± 2.3
